# Differentially Expressed Genes of Natural Killer Cells Can Distinguish Rheumatoid Arthritis Patients from Healthy Controls

**DOI:** 10.3390/genes11050492

**Published:** 2020-04-30

**Authors:** Noha Mousaad Elemam, Mahmood Yaseen Hachim, Suad Hannawi, Azzam A. Maghazachi

**Affiliations:** 1 College of Medicine and Sharjah, Institute for Medical Research, University of Sharjah, Sharjah 27272, UAE; U16101425@sharjah.ac.ae (M.Y.H.); amagazachi@sharjah.ac.ae (A.A.M.); 2 Department of Rheumatology, Ministry of Health and Prevention, Dubai 1853, UAE; suadhannawi@gmail.com

**Keywords:** rheumatoid arthritis, NK cells, biomarker, CXCL16, IL-1beta

## Abstract

Rheumatoid arthritis (RA) is one of the most prevalent autoimmune diseases, while its molecular triggers are not fully understood. A few studies have shown that natural killer (NK) cells may play either a pathogenic or a protective role in RA. In this study, we sought to explore NK cell markers that could be plausibly used in evaluating the differences among healthy controls and RA patients. Publicly available transcriptome datasets from RA patients and healthy volunteers were analyzed, in order to identify differentially expressed genes (DEGs) between 1. different immune cells as compared to NK cells, and 2. NK cells of RA patients and healthy controls. The identified DEGs were validated using 16 healthy controls and 17 RA patients. Peripheral blood mononuclear cells (PBMCs) were separated by Ficoll density gradient method, while NK cells were isolated using RosetteSep technique. RNA was extracted and gene expression was assessed using RT-qPCR. All selected genes were differentially expressed in NK cells compared to PBMCs. *CD56*, *CXCL16*, *PECAM-1*, *ITGB7*, *BTK*, *TLR10*, and *IL-1β* were significantly upregulated, while *CCL2*, *CCR4*, *RELA* and *IBTK* were downregulated in the NK cells of RA patients when compared to healthy controls. Therefore, these NK specific genes might be used as promising biomarkers for RA diagnosis.

## 1. Introduction 

Rheumatoid arthritis (RA) is the most common inflammatory arthritis disease and one of the most prevalent autoimmune disorders, affecting 1–3% of the world’s population [1]. RA is a chronic inflammatory disease, damaging the synovial lining of the joints [2]. Furthermore, it is a systemic disease where inflammation has painful and debilitating immediate effects, due to cartilage and bone erosion, corroborated with an elevated risk of cardiovascular events [3,4]. The majority of RA patients are women, where the prevalence is approximately double that in men [5], and the onset of the disease usually occurs during their 30–40 s [6].

The classification criteria for RA were set and revised by the American College of Rheumatology-European League against Rheumatism criteria (ACR-EULAR). According to the 2010 ACR-EULAR criteria, an individual is categorized as an RA patient based on a point system evaluation that depends on the count of inflamed/swollen joints, the presence of specific autoantibodies such as rheumatoid factor (RF) and/or anti-citrullinated peptide antibodies (ACPAs), acute-phase reactants and symp toms duration [7]. 

Most cell types that are associated with autoimmune diseases, such as monocytes, natural killer (NK) cells, B cells and T cells, can be collected from blood samples. It is possible that there is a similarity between the tissue-specific transcriptome expression patterns and those detected in the blood [8]. Human NK cells perform several important functions and comprise about 10–15% of total blood lymphoid cells. In the blood circulation, human NK cells are classified into two major subsets: those that express CD56, known as CD56^bright^, and those that express low CD56, known as CD56^dim^ [9,10]. Few studies have indicated that NK cells may play either a pathogenic or a protective role in RA [11,12]. Therefore, the identification of RA specific transcriptional signature in NK cells can shed some light on their molecular contribution in RA, where such a signature could facilitate the selection of biomarkers, which can be used for the early detection of RA disease. In this study, we sought to explore NK-cell-specific markers that could possibly be used to aid in distinguishing RA patients from healthy controls. 

## 2. Materials and Methods

### 2.1. In Silico Prediction of the Percentage and Status of Immune Cells in Blood of RA Patients

Whole blood transcriptomic data (GSE93272) was used to predict the percentage and status of immune cells in the blood of RA patients compared to the healthy samples using CIBERSORT, a computational method for quantifying cell fractions from sample gene expression profiles [13].

### 2.2. Using Publicly Available Data to Identify Differentially Expressed Genes (DEGs) in NK Cells

In order to understand the molecular basis of NK cells especially in RA patients, publicly available transcriptome dataset of sorted immune cells (GSE93776) was extracted using geo omnibus platform (https://www.ncbi.nlm.nih.gov/geo/). The data were analyzed using in house pipeline for normalization and filtration of the raw probes’ expression. Gene set enrichment analysis was used to identify DEGs between different immune cells as compared to NK cells. Next, DEGs between the NK cells of RA patients and healthy controls were selected, as they showed an enrichment score above the third quartile (Q3) or less than the first quartile (Q1) of all gene enrichment scores. Figure S1 illustrates a flow chart that summarizes the *in silico* approach used to obtain the DEGs of NK cells in rheumatoid arthritis.

### 2.3. RA Patients and Healthy Controls Sample Collection

Healthy controls and RA patients were recruited from the Rheumatology clinic at Al-Kuwait hospital (Dubai, United Arab Emirates). All investigations were carried out following the rules of the Declaration of Helsinki of 1975. Ethical approval was obtained from the Ministry of Health and Prevention, United Arab Emirates, (Reference no MOHAP/DXB/SUBC/No 20/2016). All healthy controls and RA patients provided written informed consent. Patients were chosen according to EULAR-ACR 2010 criteria, with the following exclusion criteria: none of the patients were pregnant, had no malignancies, or any liver impairment, renal failure or any other inflammatory arthritis or any connective tissue diseases.

### 2.4. Isolation of PBMCs and NK Cells from Peripheral Blood

From each RA patient and healthy control, 25 mL of peripheral blood were collected in sodium citrate tubes and then processed within 2 h of collection. For peripheral blood mononuclear cells (PBMCs) isolation, whole blood was layered over Histopaque-1077 (Sigma-Aldrich, Darmstadt, Germany). NK cells were isolated from whole blood by incubation with the antibody cocktail of the RosetteSep negative selection isolation kit (StemCell Technologies, Vancouver, British Columbia, Canada). The blood was layered over Histopaque-1077, and the layer containing PBMCs or NK cells was collected and washed twice with PBS. The plasma was collected and stored at −80 °C until further use. Cell pellets were then used for RNA extraction.

### 2.5. RNA Extraction, Primer Design and RT-qPCR

PBMCs and NK cell pellets were lysed using RLT lysis buffer and RNA was extracted using QIAamp RNA Blood Mini Kit (Qiagen, Hilden, Germany). The complementary DNA (cDNA) was synthesized from 200 ng of total RNA using the high-capacity reverse transcription kit (ThermoFisher Scientific, Waltham, MA, USA), according to the manufacturer’s protocol. Quantitative real time PCR was performed using 5x HOT FIREPol EvaGreen qPCR Supermix (SolisBioDyne, Tartu, Estonia) along with the primers listed in Table S1. *IFN-γ* (Hs00989291_m1) and *GAPDH* (Hs02758991_g1) were assessed using TaqMan primers along with TaqMan Gene Master Mix (Applied Biosystems, Waltham, MA, USA). All reactions were performed using Quantstudio3 system (Applied Biosystems, Waltham, MA, USA). Apart from the TaqMan primers, all other primers were designed using PrimerBlast tool by NCBI (https://www.ncbi.nlm.nih.gov/tools/primer-blast/). The resultant amplicons’ size of the qPCR was validated using agarose gel electrophoresis.

### 2.6. ELISA Quantification of Plasma CXCL16, IFN-γ and IL-1β 

Plasma proteins of CXCL16, IFN-γ and IL-1β were assessed using enzyme linked immunosorbent assay (ELISA) techniques. The kits used were human CXCL16 (DY1164), IFN-γ (DY285) and IL-1β (DY201) duosets (R&D systems, Minneapolis, MN, USA). Plasma was diluted and the protocol was followed as per the manufacturer’s instructions. Measurement of the color intensity was performed using the BioTek ELx 808 plate reader (BioTek Instruments, Winooski, VT, USA) at an absorbance wavelength of 450 nm.

### 2.7. Statistical Analysis

Statistical analyses were performed using GraphPad Prism 6 (GraphPad Software, San Diego, CA, USA). Normality test was implemented, and the significant values were generated using unpaired Student’s *t*-test or Mann–Whitney test. The data are displayed as a mean ± standard error of the mean and a *p* value < 0.05 was considered to be statistically significant. All correlation analyses were performed using nonparametric Spearman method. 

## 3. Results

### 3.1. Reduced Activated NK Cells in the Peripheral Blood of RA Patients

**Figure 1:**
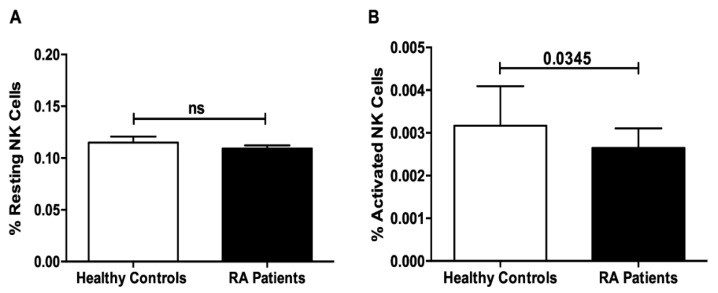
Percentage of resting (**A**) and activated (**B**) natural killer (NK) cells in peripheral blood of rheumatoid arthritis (RA) patients and healthy controls that are analyzed from publicly available whole blood transcriptomic data using *in silico* flow cytometry CIBERSOFT software.

Publicly available whole blood transcriptomic data (GSE93272) were extracted and analyzed using *in silico* flow cytometry software CIBERSORT. The percentage of each of the immune cells was compared between the peripheral blood of RA patients and healthy controls. As shown in Figure 1A, the percentage of resting NK cells in the periphery was unaltered in RA patients. In contrast, the percentage of activated NK cells was significantly decreased in the blood of RA patients when compared to healthy individuals (*p* < 0.04) (Figure 1B). 

### 3.2. Identification of Differentially Expressed Genes (DEGs) in NK Cells of RA Patients

**Figure 2:**
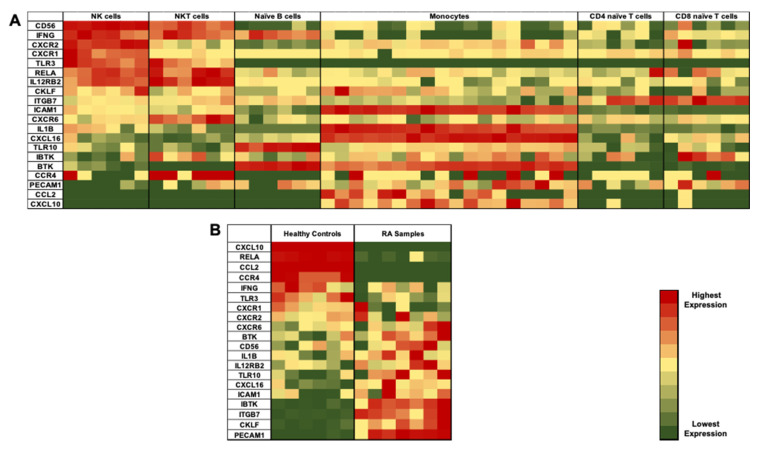
Bioinformatic analysis of publicly available transcriptome dataset from sorted immune cells revealed (**A**) list of genes that can distinguish NK cells from other immune cells present in the peripheral blood mononuclear cells (PBMCs) including natural killer T (NKT) cells, naïve B cells, monocytes, naïve CD4 and CD8 T cells (**B**) list of genes that can distinguish NK cells of RA patients from healthy controls. The red color indicates the highest fold gene expression while the green color indicates the lowest fold gene expression.

In order to investigate the molecular changes in NK cells of RA patients, publicly available transcriptome datasets (GSE93776) of sorted immune cells from RA patients and healthy volunteers were analyzed. A distinct signature of genes that might distinguish NK cells from other different immune cells was identified (Figure 2A). Next, gene set enrichment analysis (GSEA) was performed in order to select DEGs between the NK cells of RA patients and healthy controls. A comparison of DEG between controls and RA patients defined the differential expression of certain genes among the two populations (Figure 2B). These DEGs were shortlisted for further validation.

### 3.3. Validation of DEGs in Recruited Individuals’ PBMCs and NK Cells 

**Figure 3:**
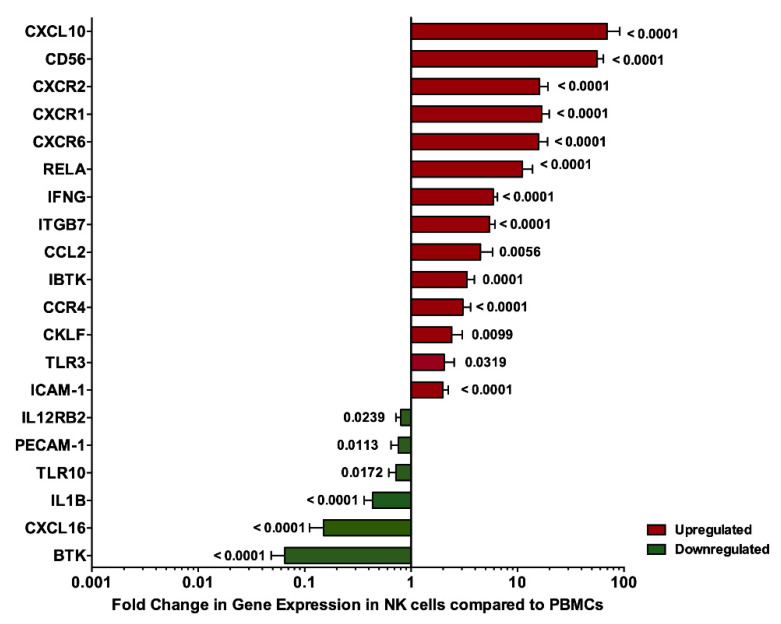
Gene expression assessed in NK cells and PBMCs of all studied samples, whether RA patients or healthy controls. *p* values are indicated for each gene when comparing NK gene expression to that of PBMCs.

PBMCs and NK cells were isolated from both RA patients and healthy controls. Validation of the DEGs was performed using RT-qPCR. As shown in Figure 3, markers were compared among NK cells and PBMCs in healthy controls or RA patients. We observed an upregulation of these genes in NK cells as compared to PBMCs: *CXCL10, CD56, CXCR2, CXCR1, CXCR6, RELA, IFN-γ, ITGB7, CCL2, IBTK, CCR4, CKLF, TLR3* and *ICAM-1*. In contrast, *IL-12RB2, PECAM-1, TLR10, IL-1β, CXCL16* and *BTK* genes were downregulated in NK cells when compared to PBMCs. This suggests that these genes can differentiate between NK cells and other cell types.

### 3.4. Gene Expression Profiling of NK Cells in RA Patients

**Figure 4:**
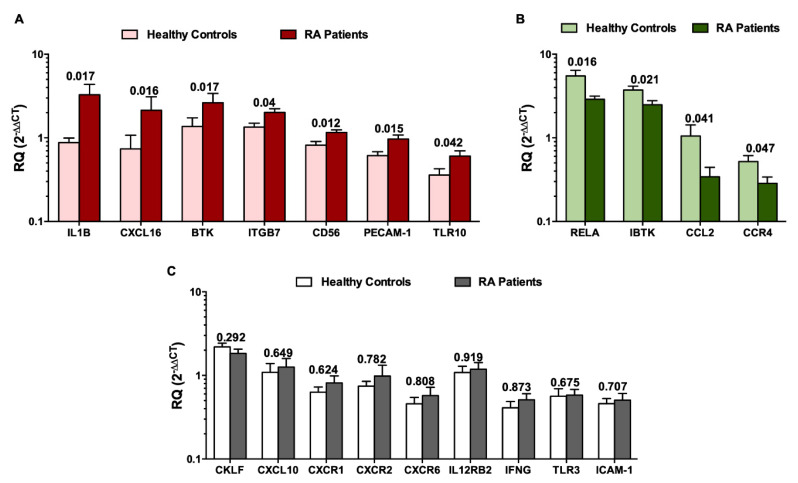
Gene expression assessed in NK cells of RA patients. (**A**) Upregulated, (**B**) Downregulated, (**C**) Non-significant gene expression when comparing RA patients to healthy controls. *p* values are indicated for each gene in comparison to healthy controls.

To demonstrate whether the genes that are up- or down-regulated in NK cells, may be utilized in order to differentiate between NK cells of RA patients and healthy controls, we investigated whether the identified 20 genes can differentiate between NK cells of RA patients when compared to healthy controls. As shown in Figure 4A, *IL-1β, CXCL16, BTK, ITGB7, PECAM-1, CD56,* and *TLR10* were significantly upregulated in NK cells of RA patients when compared to NK cells of healthy controls (Figure 4A). On the other hand, the p65 subunit of NF-κB *RELA, IBTK, CCL2* and *CCR4* showed a significant downregulation in the NK cells of RA patients (Figure 4B, *P* values indicated on each bar). The remaining genes, which include *CKLF, CXCL10, CXCR1, CXCR2, CXCR6, IL12RB2, IFNG, TLR3* and *ICAM-1,* did not show any significant differential expression between the NK cells of RA patients and healthy controls (Figure 4C).

### 3.5. Clinical Data of Recruited Healthy Controls and RA Patients and Correlation with DEGs in NK Cells 

Blood tests and clinical data of RA patients are summarized in Table S2. Out of the 17 RA patients, 65% were RF positive. It is worth mentioning that healthy controls and RA patients showed comorbidities such as dyslipidemia, diabetes, hypertension and hypothyroidism. In healthy controls, 19% had dyslipidemia, 6% had diabetes, and 13% with hypertension, while for RA patients, 18% had dyslipidemia, 35% with diabetes, 18% with hypertension and 24% with hypothyroidism. Regarding the biological treatment of RA patients, 41% of the patients did not take any biological disease-modifying anti-rheumatic drugs (bDMARDs). The remaining RA patients received either Rituxan (18%), anti-TNF therapies including Golimumab, Etanercept and Adalimumab (24%), IL-6 blocker Tocilizumab (12%) or Janus kinase (JAK) inhibitor Baricitinib (6%).

**Figure 5:**
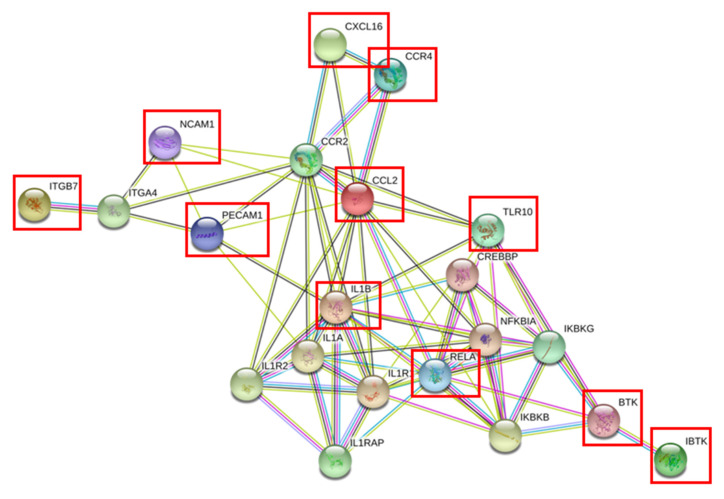
Possible common pathways that could link the 11 significant DEGs in NK cells generated by STRING software.

Correlation analysis was performed in order to investigate if the DEGs in NK cells correlate with any of the clinical parameters of the healthy controls and RA patients. Table S3 summarizes the Spearman correlation analysis executed between each of the clinical parameters and the relative quantification of each of the DEGs’ expression in NK cells. Furthermore, another Spearman correlation was performed between each of the DEGs expression with respect to the remaining genes in an attempt to identify if there is any possible link between all the DEGs. As shown in Figure S2, there was a significant correlation between the DEGs (as highlighted in yellow). This suggests that the expression of one of these genes could consequently affect the expression of the other remaining 19 genes that are part of this gene signature. This indicates that these DEGs could possibly be somehow linked to each other through a common pathway. Next, an online tool called STRING (https://string-db.org/) was utilized in order to discover which pathway could link the 11 NK significant DEGs [14]. The official symbols of these 11 genes were entered into the online tool in an attempt to find a common pathway. As shown in Figure 5, the 11 significant genes in NK cells (displayed in red squares) could possibly be linked and might share a common pathway. This link could be a direct one, such as that observed in the chemokine pathway (*CCR4, CCL2 and CXCL16*) and that with *BTK* and its inhibitor, or indirectly via other mediator signaling molecules. As suggested, the pro-inflammatory cytokine *IL-1β* is a crucial linker as it directly affects four of the significant genes that are further linked to the remaining ones. Furthermore, as listed in Table S4, the Reactome Pathways Option of STRING suggested possible pathways that these genes might share such as inflammation, TLR-signaling cascade, innate and adaptive immune system, interleukin receptors, signaling pathways, cytokines, chemokines, extracellular matrix organization and integrin cell surface interactions.

### 3.6. Plasma Cytokines and Chemokines as Potential Biomarkers

**Figure 6:**
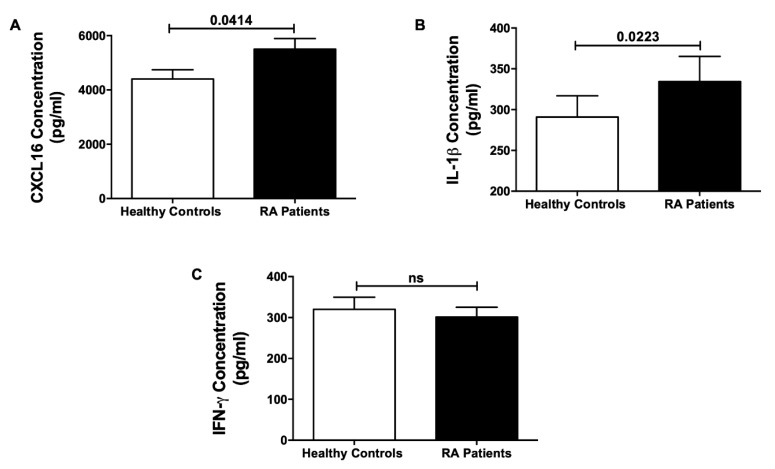
ELISA Quantification of (**A**) CXCL16 and (**B**) IL-1β and (**C**) IFN-γ in the plasma of RA patients and healthy controls.

Finally, it was important to investigate if the top two upregulated chemokines and cytokines in NK cells, i.e., CXCL16 and IL-1β, may show a similar increase in the plasma of the same RA patients examined earlier. Quantification of chemokine CXCL16 and cytokine IL-1β from the plasma of healthy controls and RA patients was measured and showed increased levels in RA patients as compared to the controls (Figure 6A,B). This is in contrast with IFN-γ, which showed no significant difference between RA patients and healthy controls (Figure 6C).

## 4. Discussion

Rheumatoid arthritis is a clinically heterogenous systemic autoimmune disease, that mainly affects synovial joints. The approach for classification of autoimmune rheumatic diseases has been previously suggested to be based on their molecular basis that could possibly lead to the discovery of novel biomarkers for patient stratification and diagnosis [15]. Hence, each disease could be represented in gene expression signature composed of multiple genes [16]. Being an autoimmune disorder, various immune cells are involved in RA, whether in the peripheral blood or residing in the synovium. Most of the cell types that are associated with autoimmune diseases, such as monocytes, NK cells, B cells and T cells, can be isolated from blood samples. It is plausible that there may be similarities between the tissue-specific (i.e., synovium) transcriptome expression patterns and those detected in the blood [8]. 

NK cells, members of innate lymphoid cells (ILCs) group 1, have been investigated in autoimmune diseases [17]. Several reports have revealed an accumulation of NK cells within inflamed joints of RA patients [18,19,20]. Upon migration into the inflamed joints, NK cells could participate in the pathogenesis of RA disease, via cytokine and chemokine production as well as through cell to cell interactions with immune and non-immune cells present in the joints [17,21]. Despite these observations, the role of NK cells in RA is still controversial as to whether it is pathogenic or protective. The pathogenic role of NK cells could be attributed to their ability to secrete proinflammatory cytokines such as TNF-α and IFN-γ, which cause the activation of other cells such as dendritic cells [22,23]. Additionally, NK cells have been described to co-stimulate B and T cells as well as trigger cytokine production by fibroblast-like synoviocytes [24]. NK cells are also able to induce osteoclast differentiation upon co-culturing with monocytes [25]. On the other hand, NK cells could possess a regulatory/protective role in RA via lysing activated macrophages and T cells [26,27]. Being the main producers of IFN-γ, NK cells have the ability to inhibit osteoclast differentiation and bone destruction as well as suppress Th17 differentiation, the major pathogenic T cell subset in RA [28]. Several studies investigated the percentages and numbers of NK cells, which was found to be decreased in the peripheral blood of RA patients [29,30,31]. In this study, we focused on the genetic expression profile of peripheral NK cells, as it has been previously suggested that systemic inflammation precedes joint inflammation [32]. Our *in silico* results showed a significant reduction in the percentage of activated NK cells in the blood of RA patients. This confirms previous observations showing an impairment of NK cell activity in RA disease [30,31,32,33,34]. 

Bioinformatic analysis of a publicly available transcriptome dataset of sorted immune cells (GSE93776) was performed in order to identify the molecular changes in NK cells of RA patients. The identified short list of genes included: a. known NK cell marker *CD56*; b. chemokine ligands *CKLF, CCL2, CXCL10* and *CXCL16*; c. chemokine receptors *CCR4*, *CXCR1, CXCR2* and *CXCR6*; d. inflammatory cytokines *IL-1β* and *IFN-γ*; e. cytokine receptors *IL12RB2*; f. Bruton’s tyrosine kinase *BTK* and its inhibitor *IBTK*; g. adhesion molecules *ITGB7, PECAM-1*, and *ICAM-1*, h. p65 subunit of NF-κB pathway *RELA*; and i. toll like receptors *TLR3* and *TLR10*. These molecules were then validated in PBMCs and NK cells in RA patients and healthy controls. We observed that the DEGs were differentially expressed in NK cells compared to PBMCs of all individuals, whether they are healthy individuals or RA patients. Out of these DEGs, 11 genes were differentially expressed in NK cells of RA patients. Moreover, the relative quantification of the DEGs showed a correlation with the clinical parameters. Additionally, a correlation matrix was generated between each of the DEGs, with respect to the remaining genes, where it showed some significant correlations, highlighting that they might share some common pathways. This was further supported by the *in silico* tool STRING, that showed possible direct and indirect pathways between the 11 significantly reported genes. These shared pathways include inflammation, TLR signaling cascade, innate and adaptive immune system, interleukin receptors, signaling pathways, cytokines, chemokines, extracellular matrix organization and integrin cell surface interactions. The well-known NK marker *CD56(NCAM-1)* was upregulated in the NK cells of RA patients, supporting previous findings showing the expansion of CD56^bright^ NK cells in various autoimmune diseases [35,36]. It has been reported that there was an elevation in the number of CD56^bright^ NK cells in RA patients, that are negative for RF and anti-CCP antibodies [29]. NK cells present in the synovium of RA patients have been previously found to highly express CD56 [19]. 

Bruton tyrosine kinase (BTK) is a key intracellular enzyme, known to play a crucial role during the maturation of NK cells by inducing IFN-γ production and cytolytic activity [37]. Furthermore, BTK was reported to play a crucial role in bone resorption of RA patients, via regulating the signal transduction through a receptor activator of nuclear factor kappa-B (RANK), the receptor for RANK ligand (RANK-L) that drives osteoclast differentiation and activation [38,39]. In this study, we demonstrated that *BTK* is upregulated in NK cells of RA patients, while its inhibitor *IBTK* was found to be downregulated in the NK cells of RA individuals. BTK was found to affect the NF-κB signaling pathway [37,40]. Similarly, the p65 subunit of NF-κB *RELA* was downregulated in NK cells of RA patients, displaying a less activated phenotype. Collectively, our observations suggest that NK cell activation in RA patients is impaired.

A vital pro-inflammatory cytokine *IL-1β* was upregulated in the NK cells of RA patients. This is similar to the study of Chan et al., where they suggested that activated NK cells upon coculture with fibroblast like synoviocytes secrete IL-1β [24]. Further, they demonstrated that IL-1β secretion is higher in RA patients when compared to osteoarthritis (OA) individuals [24]. On the contrary, the primary cytokine secreted by NK cells, i.e., *IFN-γ,* as well as the cytokine receptor component *IL12RB2,* were found to be unaltered in RA disease. This may indicate that “IFN-γ producing NK cells” are mainly found in the RA synovium, which may consequently, activate macrophages and osteoclasts [41]. As suggested by the online tool STRING, *IL-1β* was the most crucial marker, being a central player that may directly affect four of the DEGs and hence have an indirect effect on the remaining ones (Figure 5).

Two major key players in the migration of lymphocytes to the RA synovium are adhesion molecules and chemokines. Several studies have highlighted that ICAM-1 is upregulated in RA disease as it aids in the rolling and adhesion of leukocytes during the migration process. However, this does not seem to be the case in NK cells, where *ICAM-1* expression was unchanged. Nevertheless, another adhesion molecule, *PECAM-1,* was significantly upregulated in the NK cells of RA patients. This finding is in line with a similar study reporting high levels of serum-soluble PECAM-1 in RA patients [42]. In NK cells, PECAM-1 was found to play a crucial role in NK cell extravasation into tissues and inflammatory sites [43]. Moreover, the ligation of PECAM-1 on the surface of NK cells was found to activate β integrins [43]. Hence, this could explain the concomitant increase in *integrin β7 (ITGB7)* on the NK cells of RA patients. 

Chemokines play a major role in the migration of various cell types into inflammatory sites in arthritis [44]. CXCR1 and CXCR2 have been shown to be abundantly expressed on neutrophils, where they promote their migration into the joints, leading to the pathogenesis of RA disease [45]. However, these receptors do not seem to be important for NK cells in RA patients. It was previously reported that RA patients exhibited increased levels of CCL2 and CXCL10 in the plasma as well as the synovial fluid [13,46,47,48]. Furthermore, CCL2 and CXCL10 are associated with immune cells’ infiltration into the joints and disease activity scores, respectively [49,50]. The expression of these chemokines in NK cells seems to be quite different, where *CCL2* is downregulated in RA patients while *CXCL10* expression is unaltered. Another close member to the chemokines is the chemokine-like factor (CKLF), a functional ligand for CCR4 [51], that has been found to be upregulated on activated T cells in RA disease [52]. However, *CKLF* was not found to be differentially expressed in NK cells while its receptor *CCR4* was found to be downregulated in the NK cells of RA patients. *CCR4* expression has not been previously investigated in the NK cells of RA patients, although it was observed that it is highly expressed on peripheral and synovial T cells in RA disease [53,54]. The CXCR6/CXCL16 axis was found to play a role in the pathogenesis of RA and contribute to joint inflammation [55]. CXCR6 is highly expressed on memory T cells in the RA synovium compared to OA [56,57]. In our study, we observed that, despite the expression of *CXCR6* on NK cells, this expression remains unchanged in RA disease. On the other hand, its ligand CXCL16 was known to be enhanced locally in RA synovium [56,57,58]. *CXCL16* gene was downregulated in NK cells when compared to all PBMCs. The higher expression of CXCL16 in PBMCs may be attributed to its release by macrophages [55]. Despite not being highly expressed by NK cells, *CXCL16* expression was upregulated in the NK cells of RA patients, thus highlighting a potential pathogenic role in the NK cells of RA patients.

Innate immune cells such as NK cells are known to express TLRs [59], that activate and mediate immune responses leading to the production of pro-inflammatory cytokines. Endosomal TLR3 has been previously investigated in RA disease, where it was highly expressed in RA synovial tissues [60,61,62]. We report that *TLR3* expression was unaffected, while the expression of the transmembrane *TLR10* was upregulated in the NK cells of RA patients. A previous study highlighted an elevated TLR10 in B cell subsets that correlates with RA disease activity [63]. 

Out of the seven upregulated DEGs in the NK cells of RA patients, it was crucial to look for a potential secreted biomarker in the plasma of these individuals, and hence the cytokine IL-1β and chemokine CXCL16 were investigated. Indeed, IL-1β was significantly elevated in the plasma of RA patients, supporting previous data where elevated plasma IL-1 concentrations were observed in RA patients [64]. CXCL16 plasma levels were also higher in RA patients, similar to previous reports [65,66]. 

This is the first study to report the potential impact of these genes in NK cells of RA disease. However, the limitations of this study could be that further in-depth investigation on the protein level should be done in order to fully understand the function of these DEGs and whether they affect the role of NK cells, either protective or pathogenic, in RA. Additionally, another limitation could be the sample size included in the study. Future studies could possibly have a bigger sample size that should enable further classification of RA patients according to their status (i.e., treatment naïve) or the type of therapy utilized. In conclusion, this study highlights that NK cells play a crucial role and are affected in RA disease. Furthermore, a gene signature profile of these NK cells could shed some light on the potential impact of such DEGs [67]. Consequently, the 11 genes *CXCL16, IL-1β, CD56, PECAM-1, ITGB7, TLR10, IBTK, BTK, CCL2, CCR4,* and *RELA* can be used in differentiating RA patients form healthy state.

## Supplementary Materials

The following are available online at https://www.mdpi.com/2073-4425/11/5/492/s1, Figure S1: Flow chart summarizing the in-silico approach used in the study in order to obtain the DEGs of NK cells in RA, Figure S2: Spearman correlation matrix between each of the DEGs in comparison to the rest of the genes in NK cells of RA patients and healthy controls, Table S1: List of primers for assessment of gene expression using real time qPCR, Table S2: Summary of Clinical Data of Recruited Healthy Controls and RA Patients, Table S3: Significant correlation analyses between DEGs in NK cells and clinical parameters of healthy controls and RA patients, Table S4: Pathways shared by the 11 significant selected genes in Reactome Pathways Option of STRING.

## Abbreviations

18S	18S ribosomal RNA
ACPAs	Anti-citrullinated peptide antibodies
ACR-EULAR.	American college of rheumatology-european league against rheumatism criteria
Anti-CCP	Anti-citrullinated peptide
bDMARDs	Biological disease modifying anti-rheumatic drugs
BTK	Bruton’s tyrosine kinase
CCL	C chemokine ligand
CCR	C chemokine receptor
CD56	Cluster of differentiation 56
CIA	Collagen induced arthritis
CKLF	Chemokine like factor
CRP	C- reactive peptide
CXCL	CX chemokine ligand
CXCR	CX chemokine receptor
DEGs	Differentially expressed genes
ELISA	Enzyme linked immunosorbent assay
ESR	Erythrocyte sedimentation rate
GAPDH	Glyceraldehyde 3-phosphate dehydrogenase
GSEA	Gene set enrichment analysis
IBTK	Inhibitor of Bruton’s tyrosine kinase
ICAM-1	Intercellular adhesion molecule-1
IFNG	Interferon gamma
IL-	Interleukin
ILC	Innate lymphoid cells
ITGB7	Integrin beta 7
JAK	Janus kinase
MS	Multiple sclerosis
NK cells	Natural killer cells
OA	Osteoarthritis
PBMCs	Peripheral blood mononuclear cells
PECAM-1	Platelet endothelial cell adhesion molecule-1
RA	Rheumatoid arthritis
RANK	Receptor activator of nuclear factor kappa-B
RELA	p65 subunit of NF-kB
RF	Rheumatoid factor
RNA	Ribonucleic acid
SEM	Standard error of mean
SLE	Systemic lupus erythematosus
TLR	Toll like receptor
TNF	Tumor necrosis factor
WBC	White blood cells
