# Supplementary material for: Differentially Expressed Genes of Natural Killer Cells Can Distinguish Rheumatoid Arthritis Patients from Healthy Controls
Source: Genes (Basel). 2020 Apr 30;11(5):492. doi: 10.3390/genes11050492 (PMC7290970; doi:10.3390/genes11050492)
Supplement: Supplementary file 1 [file genes-11-00492-s001.pdf]

Supplementary Tables and Figures

Table S1. *List of primers for assessment of gene expression using real time qPCR.*

Gene Name	Primer Sequence	Amplicon Size (bp)
**CXCL10**	**F:** AAGGAATCTTTCTGCTTTGG<br>**R:** ACCTTCCTGTATGTGTTTGG	184
**RELA**	**F:** TAGAAGAGCAGCGTGGGGA<br>**R:** AGAGTTTCGGTTCACTCGGC	176
**CCL2**	**F:** CAGCCAGATGCAATCAATGCC<br>**R:** TGGAATCCTGAACCCACTTCT	190
**CCR4**	**F:** GAAAGTATCCCCAAGCCTTGC<br>**R:** AAGGGAGGGAAAACACGAAGA	214
**IBTK**	**F:** AAGTCTGGTAAGACCAATTCTGTG<br>**R:** CGGTTTGACTTTCGCCTCAAT	156
**ITGB7**	**F:** GTGGAAACTAAAGCCCAGAGAG<br>**R:** CCGAGATCCCAAGCCGTAG	122
**CKLF**	**F:** TATCACTGGATTTGAAGTCACCG<br>**R:** GCCAACACAGATACGATGAGC	153
**PECAM-1**	**F:** CCCAGCCCAGGATTTCTTAT<br>**R:** ACCGCAGGATCATTTGAGTT	163
**CXCL16**	**F:** CCCGCCATCGGTTCAGTTC<br>**R:** CCCCGAGTAAGCATGTCCAC	181
**TLR3**	**F:** GTCCCAAGCCTTCAACGACT<br>**R:** GTTTGCGTGTTTCCAGAGCC	216
**BTK**	**F:** GCTCAAAAACGTAATCCGGTACA<br>**R:** GTCTTCCGGTGAGAACTCCC	171
**ICAM1**	**F:** GGTAGCAGCCGCAGTCATAA<br>**R:** GATAGGTTCAGGGAGGCGTG	149
**TLR10**	**F:** GATTTACTCTGGGACGACCTTTT<br>**R:** GTCAAGATAAGCCTTACCACCAA	108
**IL1B**	**F:** AGCTACGAATCTCCGACCAC<br>**R:** CGTTATCCCATGTGTCGAAGAA	186
**IL12RB2**	**F:** AGGACGAGACACCCACTTATAC<br>**R:** ATTGACAGCAGTAACCTTGGC	172
**CXCR6**	**F:** ATGTGTTCCTGGTGAACCTA<br>**R:** GGCCTTAACCACTACAATGA	203
**CXCR2**	**F:** GACAAATCTCCACCTTCAGA<br>**R:** ATGAAACAACCTTGACGATG	224
**CXCR1**	**F:** TGGGGACTGTCTATGAATCTGT<br>**R:** GCAACACCATCCGCCATTTT	127
**CD56**	**F:** TGTCCGATTCATAGTCCTGTCC<br>**R:** CTCACAGCGATAAGTGCCCTC	82

**18S**	**F:** TGACTCAACACGGGAAACC<br>**R:** TCGCTCCACCAACTAAGAAC	114

Table S2. Summary of Clinical Data of Recruited Healthy Controls and RA Patients

WBC: White blood cells, RBC: red blood cells, HCT: Hematocrit, MCV: mean corpuscular volume, MCH: mean corpuscular hemoglobin, MCHC: mean corpuscular hemoglobin concentration, RDW: red blood cell distribution width, MPV: mean platelet volume, PDW: platelet distribution width, HbA1c: Hemoglobin A1c, GFR: Glomerular filtration rate, HDL: high-density lipoprotein, LDL: low-density lipoprotein, TSH: Thyroid-stimulating hormone, TPO: Thyroid peroxidase, CRP: C-reactive protein, BSA: Body Surface Area, BMI: Body Mass Index, RF: Rheumatoid Factor

	**Healthy Controls**			**Rheumatoid Arthritis Patients**		
**Parameters**	**Range**	**Mean ± SD**	**Mean ± SEM**	**Range**	**Mean ± SD**	**Mean ± SEM**
**Age**	23-70	45.81 ± 10.07	45.81 ± 2.517	27-89	53.76 ± 14.83	53.76 ± 3.597
**WBC**	2.61-7.84	5.449 ± 1.4	5.449 ± 0.35	3.87-13.86	6.901 ± 2.343	6.901 ± 0.5684
**RBC**	4.02-5.42	4.639 ± 0.487	4.639 ± 0.1218	3.45-5.58	4.664 ± 0.5589	4.664 ± 0.1356
**Hemoglobin**	11.3-13.6	12.68 ± 0.7123	12.68 ± 0.1781	10.4-16.4	12.95 ± 1.341	12.95 ± 0.3253
**HCT**	33.9-41.4	37.77 ± 1.755	37.77 ± 0.4388	32.6-49.1	38.92 ± 4.027	38.92 ± 0.9767
**MCV**	67.6-95.7	82.06 ± 7.722	82.06 ± 1.931	73.1-101.5	83.88 ± 6.773	83.88 ± 1.643
**MCH**	21.8-32.3	27.62 ± 3.306	27.62 ± 0.8265	23.4-34.2	27.95 ± 2.666	27.95 ± 0.6467
**MCHC**	30.9-36.4	33.58 ± 1.392	33.58 ± 0.3481	31.2-35.8	33.29 ± 1.134	33.29 ± 0.275
**RDW**	12-19.9	13.74 ± 1.945	13.74 ± 0.4862	12.5-20	14.73 ± 1.697	14.73 ± 0.4116
**Platelet**	189-363	279.9 ± 49.36	279.9 ± 12.34	191-450	300.5 ± 74.45	300.5 ± 18.06
**MPV**	6.4-10.9	8.338 ± 1.079	8.338 ± 0.2699	6.7-13.5	8.376 ± 1.717	8.376 ± 0.4164
**% Neutrophils**	32-67.8	51.52 ± 8.479	51.52 ± 2.12	26-88.5	55.5 ± 15.45	55.5 ± 3.746
**% Lymphocytes**	25-53	37.62 ± 7.579	37.62 ± 1.895	9.7-54.6	32.85 ± 11.91	32.85 ± 2.889
**% Monocytes**	3.4-10.1	5.969 ± 1.872	5.969 ± 0.4679	1.8-12.2	6.029 ± 2.531	6.029 ± 0.6138
**% Eosinophils**	0.8-6.1	2.925 ± 1.575	2.925 ± 0.3938	0.1-11.6	3.413 ± 2.8	3.413 ± 0.7
**% Basophils**	0.1-1.1	0.5313 ± 0.2983	0.5313 ± 0.07456	0.1-1.5	0.5875 ± 0.3757	0.5875 ± 0.0939
**Absolute Neutrophils**	1.23-4.15	2.84 ± 0.9821	2.84 ± 0.2455	1.43-11.04	3.964 ± 2.448	3.964 ± 0.612
**Absolute Lymphocytes**	1.11-2.8	2.012 ± 0.5409	2.012 ± 0.1352	0.76-3.79	2.143 ± 0.7916	2.143 ± 0.1979
**Absolute Monocytes**	0.16-0.69	0.3288 ± 0.151	0.3288 ± 0.03775	0.14-0.6	0.3763 ± 0.1256	0.3763 ± 0.0314
**Absolute Eosinophils**	0.04-0.4	0.1525 ± 0.1075	0.1525 ± 0.02687	0.08-0.79	0.2386 ± 0.1835	0.2386 ± 0.0491

**Absolute Basophils**	0.01-0.05	0.02375 ± 0.0115	0.02375 ± 0.00287	0.01-0.11	0.036 ± 0.02586	0.036 ± 0.00668
**PDW**	9.4-66.9	41.39 ± 19.86	41.39 ± 4.965	9.7-69.4	40.19 ± 17.5	40.19 ± 4.376
**Erythrocyte Sedimentation Rate (ESR)**	6-50	20.69 ± 14	20.69 ± 3.884	6-80	33.76 ± 21.84	33.76 ± 5.298
**Sodium**	131-143	139.1 ± 3.125	139.1 ± 0.8351	132-143	138.5 ± 3.184	138.5 ± 0.7723
**Creatinine**	43-91	59.5 ± 12.68	59.5 ± 3.17	31-111	62.59 ± 20.34	62.59 ± 4.933
**Uric acid**	4.6-350	241.3 ± 81.8	241.3 ± 21.86	145-508	275.5 ± 88.64	275.5 ± 22.89
**Fasting Glucose**	4.9-6	5.375 ± 0.3732	5.375 ± 0.1319	6-8.5	6.9 ± 1.389	6.9 ± 0.8021
**Glucose**	4-7.8	5.558 ± 1.039	5.558 ± 0.3134	5-29.1	8.121 ± 6.164	8.121 ± 1.647
**HbA1c**	4.8-6	5.414 ± 0.414	5.414 ± 0.1565	4.9-10.4	6.615 ± 1.521	6.615 ± 0.4218
**Urea level**	2.3-8	4.231 ± 1.894	4.231 ± 0.5253	2.7-9.1	4.724 ± 1.796	4.724 ± 0.4356
**GFR**	55-119	101.4 ± 21.51	101.4 ± 7.604	39-186.1	98.88 ± 31.22	98.88 ± 7.805
**Total protein**	70-92	78.69 ± 6.183	78.69 ± 1.715	68-85	75.94 ± 4.582	75.94 ± 1.146
**Albumin**	33.1-45	38.86 ± 3.821	38.86 ± 1.06	29.2-38.9	34.16 ± 3.208	34.16 ± 0.802
**Total Bilirubin**	6.6-18.4	9.731 ± 3.216	9.731 ± 0.8919	4.2-14.9	8.606 ± 3.568	8.606 ± 0.8921
**Ferritin**	10-162	67.88 ± 51.2	67.88 ± 18.1	9-66	27.9 ± 18.26	27.9 ± 5.774
**Iron**	8.4-20.3	14.24 ± 4.837	14.24 ± 1.71	2.9-27.7	13.24 ± 8.423	13.24 ± 2.664
**Transferrin**	211-339	254 ± 46.83	254 ± 19.12	216-336	257.2 ± 41.47	257.2 ± 13.11
**Cholesterol**	3.46-7.82	5.344 ± 1.198	5.344 ± 0.3094	4-7.2	5.013 ± 0.9165	5.013 ± 0.2366
**Triglycerides**	0.57-2	1.069 ± 0.4152	1.069 ± 0.1072	0.43-3.82	1.508 ± 0.9503	1.508 ± 0.2454
**HDL**	0.9-1.87	1.424 ± 0.2761	1.424 ± 0.07129	0.95-2.11	1.399 ± 0.3633	1.399 ± 0.09379
**LDL**	1.74-5.83	3.539 ± 1.179	3.539 ± 0.3045	0.26-3.64	2.655 ± 0.8625	2.655 ± 0.2227
**Vitamin D**	30.47-100.8	55.01 ± 19.55	55.01 ± 5.047	17.82-130.7	56.3 ± 32.03	56.3 ± 8.884
**Complement 3**	97-196	137.7 ± 34.07	137.7 ± 13.91	85-202	153.7 ± 42.81	153.7 ± 17.48
**Complement 4**	11-36	26 ± 8.408	26 ± 3.432	19.9-44.2	31.83 ± 9.008	31.83 ± 3.677
**T4 free**	11.95-17.46	15.31 ± 1.887	15.31 ± 0.4871	7.67-25.64	15.63 ± 4.008	15.63 ± 1.002
**T3 free**	3.59-4.93	4.304 ± 0.5167	4.304 ± 0.1722	3.17-5.25	4.259 ± 0.6329	4.259 ± 0.2002
**TSH**	0.66-3.29	1.581 ± 0.7203	1.581 ± 0.1801	0.16-10.51	2.56 ± 2.774	2.56 ± 0.6728
**TPO Antibody**	6.6-1300	192.8 ± 406.3	192.8 ± 128.5	6.4-316.9	97.59 ± 114.8	97.59 ± 38.28

**Thryoglobin Antibody**	10-261.3	62.21 ± 85.43	62.21 ± 27.02	12-1900	306.5 ± 555.4	306.5 ± 167.5
**Thryglobulin**	1.74-164	35.63 ± 58.04	35.63 ± 21.94	0.3-405.4	70.27 ± 164.2	70.27 ± 67.04
**CRP**	0-15.4	2.846 ± 4.119	2.846 ± 1.142	0.1-94.8	13.83 ± 23.07	13.83 ± 5.595
**Height**	149-169	157.1 ± 6.26	157.1 ± 1.565	142-168	154.8 ± 5.747	154.8 ± 1.394
**Weight**	43-86	69.09 ± 12.16	69.09 ± 3.041	47.8-112	76.29 ± 16.41	76.29 ± 3.981
**BSA**	1.34-1.99	1.729 ± 0.1674	1.729 ± 0.04184	1.44-2.2	1.802 ± 0.1962	1.802 ± 0.04758
**BMI**	19-36	28.19 ± 5.18	28.19 ± 1.295	20-47	31.87 ± 7.454	31.87 ± 1.808
**Systolic Blood Pressure**	98-148	122.1 ± 15.15	122.1 ± 3.786	114-166	134.6 ± 15.64	134.6 ± 3.792
**Diastolic Blood Pressure**	52-84	69.69 ± 8.987	69.69 ± 2.247	50-94	78.18 ± 9.697	78.18 ± 2.352
**RF**	-	-	-	0.01-400 (65% Positive)	98.45 ± 140.2	98.45 ± 42.26

Table S3. *Significant correlation analyses between DEGs in NK cells and clinical parameters of healthy controls and RA patients*

Gene Name	Clinical Parameter	P value	r value
**CD56**	WBC	0.0179	-0.4098
	Absolute Lymphocytes	0.0391	-0.3665
**PECAM-1**	CRP	0.0167	0.4337
**ITGB7**	WBC	0.0435	-0.3536
**IBTK**	WBC	0.0138	-0.4245
**TLR10**	Percent Lymphocytes	0.0355	-0.3673
**IL12RB2**	Absolute Lymphocytes	0.0118	-0.4399
	ESR	0.0368	-0.3828
**CXCR1**	Percent Lymphocytes	0.0050	-0.4770
	Absolute Lymphocytes	0.0038	-0.4974
**CXCR2**	Percent Lymphocytes	0.0158	-0.4168
	Absolute Lymphocytes	0.0013	-0.5427
**CXCR6**	Percent Lymphocytes	0.0218	-0.3981
	Absolute Lymphocytes	0.0272	-0.3904
**CCL2**	ESR	0.0221	-0.4162
	CRP	0.0135	-0.4459
**CCR4**	Percent Lymphocytes	0.0417	-0.3565

Table S4. Pathways shared by the 11 significant selected genes in Reactome Pathways Option of STRING.

Description	Observed gene count	Background gene count	False discovery rate	Matching proteins in your network (IDs)	Matching proteins in your network (labels)
Immune System	8	1925	4.60E-05	ENSP00000225831,ENSP00000263341,ENSP00000267082,ENSP00000308925,ENSP00000384273,ENSP00000457421,ENSP00000480132,ENSP00000483570	BTK,CCL2,IL1B,ITGB7,NCAM1,PECAM1,RELA,TLR10
Diseases associated with the TLR signaling cascade	3	24	4.60E-05	ENSP00000308925,ENSP00000384273,ENSP00000483570	BTK,RELA,TLR10
CLEC7A/inflammasome pathway	2	6	0.00024	ENSP00000263341,ENSP00000384273	IL1B,RELA
Interleukin-1 processing	2	7	0.00025	ENSP00000263341,ENSP00000384273	IL1B,RELA
Toll-like Receptor Cascades	3	151	0.0015	ENSP00000308925,ENSP00000384273,ENSP00000483570	BTK,RELA,TLR10
Innate Immune System	5	1012	0.0023	ENSP00000263341,ENSP00000308925,ENSP00000384273,ENSP00000457421,ENSP00000483570	BTK,IL1B,PECAM1,RELA,TLR10
Cytokine Signaling in Immune system	4	654	0.0046	ENSP00000225831,ENSP00000263341,ENSP00000384273,ENSP00000480132	CCL2,IL1B,NCAM1,RELA
Chemokine receptors bind chemokines	2	48	0.0046	ENSP00000293778,ENSP00000332659	CCR4,CXCL16
Interleukin-10 signaling	2	45	0.0046	ENSP00000225831,ENSP00000263341	CCL2,IL1B
Extracellular matrix organization	3	298	0.0059	ENSP00000267082,ENSP00000457421,ENSP00000480132	ITGB7,NCAM1,PECAM1
MyD88:MAL(TIRAP) cascade	2	94	0.01	ENSP00000384273,ENSP00000483570	BTK,RELA

initiated on plasma membrane					
Integrin cell surface interactions	2	83	0.01	ENSP00000267082,ENSP00000457421	ITGB7,PECAM1
Signaling by Interleukins	3	439	0.01	ENSP00000225831,ENSP00000263341,ENSP00000384273	CCL2,IL1B,RELA
Interleukin-4 and Interleukin-13 signaling	2	106	0.01	ENSP00000225831,ENSP00000263341	CCL2,IL1B
Interleukin-1 signaling	2	98	0.01	ENSP00000263341,ENSP00000384273	IL1B,RELA
MyD88 cascade initiated on plasma membrane	2	84	0.01	ENSP00000308925,ENSP00000384273	RELA,TLR10
Signaling by the B Cell Receptor (BCR)	2	107	0.01	ENSP00000384273,ENSP00000483570	BTK,RELA
Fc epsilon receptor (FCERI) signaling	2	126	0.0108	ENSP00000384273,ENSP00000483570	BTK,RELA
Adaptive Immune System	3	733	0.0279	ENSP00000267082,ENSP00000384273,ENSP00000483570	BTK,ITGB7,RELA
Signal Transduction	5	2605	0.0373	ENSP00000293778,ENSP00000332659,ENSP00000384273,ENSP00000480132,ENSP00000483570	BTK,CCR4,CXCL16,NCAM1,RELA

Figure S1. *Flow chart summarizing the in-silico approach used in the study in order to obtain the DEGs of NK cells in RA.*

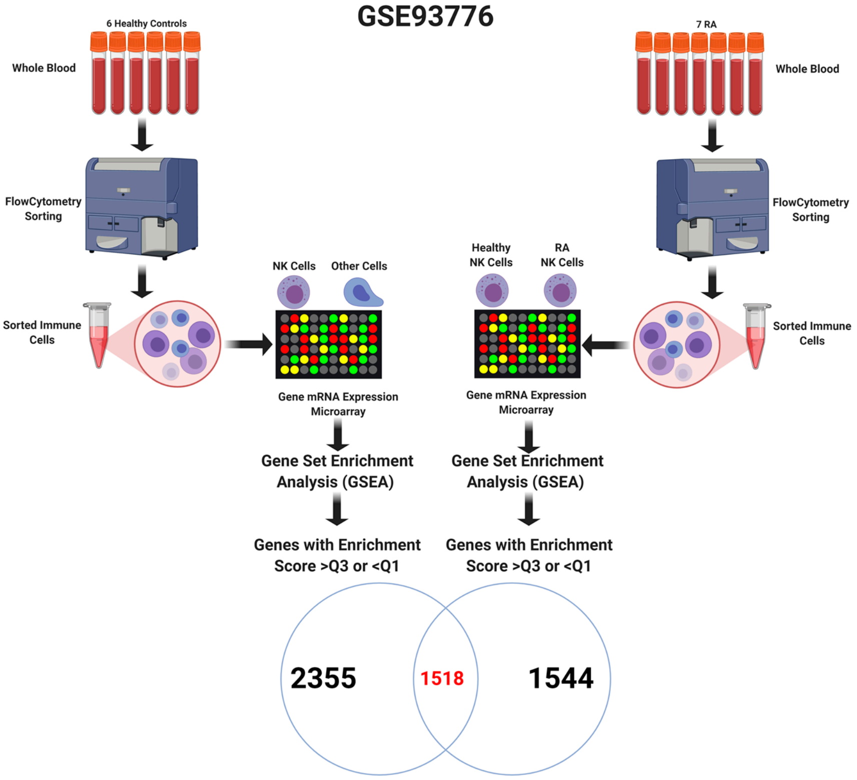

Figure S2. *Spearman correlation matrix between each of the DEGs in comparison to the rest of the genes in NK cells of RA patients and healthy controls.*

Genes	CKLF	CXCL16	CD56	CCR4	PECAM-1	IBTK	ITGB7	CCL2	CXCL10	RELA	CXCR6	CXCR1	CXCR2	TLR3	ICAM	BTK	IL12RB2	TLR10	IL1B	IFNG
CKLF		0.055	0.026	-0.363	-0.217	0.319	0.105	0.153	-0.278	0.361	-0.258	-0.218	-0.170	-0.040	-0.157	0.461	0.149	-0.077	-0.142	0.390
CXCL16	0.055		0.147	0.016	0.480	0.028	0.076	-0.200	0.120	-0.056	0.100	0.165	0.120	0.266	0.070	0.611	0.211	0.266	0.158	0.136
CD56	0.026	0.147		0.685	0.507	0.567	0.623	0.245	0.691	0.286	0.737	0.755	0.650	0.538	0.619	0.133	0.365	0.581	0.223	-0.098
CCR4	-0.363	0.016	0.685		0.569	0.095	0.255	0.411	0.884	-0.189	0.904	0.899	0.783	0.596	0.771	-0.014	0.185	0.663	0.554	-0.304
PECAM-1	-0.217	0.480	0.507	0.569		-0.024	0.378	-0.048	0.501	-0.157	0.471	0.572	0.334	0.455	0.451	0.396	0.194	0.566	0.543	-0.162
IBTK	0.319	0.028	0.567	0.095	-0.024		0.484	0.111	0.249	0.677	0.250	0.229	0.276	0.228	0.209	0.087	0.398	0.262	-0.200	0.269
ITGB7	0.105	0.076	0.623	0.255	0.378	0.484		-0.195	0.256	0.452	0.257	0.367	0.080	0.295	0.311	0.121	0.285	0.334	0.212	0.246
CCL2	0.153	-0.200	0.245	0.411	-0.048	0.111	-0.195		0.371	-0.038	0.497	0.409	0.648	0.204	0.295	-0.046	0.214	0.239	0.081	-0.176
CXCL10	-0.278	0.120	0.691	0.884	0.501	0.249	0.256	0.371		-0.019	0.859	0.766	0.808	0.512	0.755	0.060	0.313	0.610	0.476	-0.388
RELA	0.361	-0.056	0.286	-0.189	-0.157	0.677	0.452	-0.038	-0.019		-0.130	-0.157	-0.075	0.021	-0.032	-0.022	0.338	-0.071	-0.332	0.222
CXCR6	-0.258	0.100	0.737	0.904	0.471	0.250	0.257	0.497	0.859	-0.130		0.901	0.904	0.699	0.822	0.037	0.366	0.770	0.475	-0.339
CXCR1	-0.218	0.165	0.755	0.899	0.572	0.229	0.367	0.409	0.766	-0.157	0.901		0.805	0.657	0.735	0.124	0.244	0.732	0.531	-0.156
CXCR2	-0.170	0.120	0.650	0.783	0.334	0.276	0.080	0.648	0.808	-0.075	0.904	0.805		0.613	0.743	0.078	0.408	0.652	0.363	-0.325
TLR3	-0.040	0.266	0.538	0.596	0.455	0.228	0.295	0.204	0.512	0.021	0.699	0.657	0.613		0.826	0.312	0.489	0.886	0.521	-0.095
ICAM	-0.157	0.070	0.619	0.771	0.451	0.209	0.311	0.295	0.755	-0.032	0.822	0.735	0.743	0.826		0.112	0.423	0.872	0.625	-0.324
BTK	0.461	0.611	0.133	-0.014	0.396	0.087	0.121	-0.046	0.060	-0.022	0.037	0.124	0.078	0.312	0.112		0.226	0.340	0.326	0.299
IL12RB2	0.149	0.211	0.365	0.185	0.194	0.398	0.285	0.214	0.313	0.338	0.366	0.244	0.408	0.489	0.423	0.226		0.599	0.093	-0.310
TLR10	-0.077	0.266	0.581	0.663	0.566	0.262	0.334	0.239	0.610	-0.071	0.770	0.732	0.652	0.886	0.872	0.340	0.599		0.550	-0.237
IL1B	-0.142	0.158	0.223	0.554	0.543	-0.200	0.212	0.081	0.476	-0.332	0.475	0.531	0.363	0.521	0.625	0.326	0.093	0.550		-0.196
IFNG	0.390	0.136	-0.098	-0.304	-0.162	0.269	0.246	-0.176	-0.388	0.222	-0.339	-0.156	-0.325	-0.095	-0.324	0.299	-0.310	-0.237	-0.196	
